# Prevalence of Front-of-Pack Warning Signs among Commercial Complementary Foods in Seven High and Upper Middle-Income Countries

**DOI:** 10.3390/nu15071629

**Published:** 2023-03-27

**Authors:** Eleonora Bassetti, Asha Khosravi, Alissa M. Pries

**Affiliations:** Helen Keller International, New York, NY 10017, USA

**Keywords:** complementary foods, nutrient profiling, infant and young child feeding, food labelling/methods, food labelling/standards, food industry

## Abstract

Front-of-pack nutrition labelling (FOPNL) can provide a mechanism for communicating the nutritional quality of commercially produced complementary foods (CPCF) to caregivers. To better understand the role FOPNL may provide for CPCF, this study aimed to evaluate CPCFs against national and global FOPNL systems to determine the proportion that would warrant warning or traffic light signs for high levels of concerning nutrients. A cross-sectional assessment was conducted to evaluate the levels of selected nutrients in CPCF available in Australia (*n* = 266), Brazil (*n* = 41), Chile (*n* = 73), Mexico (*n* = 164), the United States (*n* = 562), the United Arab Emirates (*n* = 135), and the United Kingdom (*n* = 643). The analysis was based on national FOPNL systems and the WHO Regional Office for Europe CPCF nutrient profiling model’s ‘high sugar’ FOPNL warning. A substantial proportion of CPCFs contained excessive levels of total sugar, total fat or saturated fat that would warrant a red/amber traffic light or warning sign on product labels. Additionally, the high prevalence of added sugars and sweeteners identified in CPCFs was concerning. Based on these findings, the use of FOPNL among CPCFs could be beneficial to communicate the nutritional quality of these products to caregivers and trigger the reformulation of CPCFs with inappropriate nutrient profiles.

## 1. Introduction

Sales of commercially produced complementary foods (CPCFs) have increased worldwide [1], as has the debate on the role of these products in diets of children 6–36 months of age [1,2,3,4]. CPCFs may have practical advantages and serve as a source of energy and nutrients for older infants and young children (IYC). However, some CPCFs contain high levels of nutrients of public health concern, with recent studies showing excessive sugar and sodium content in many of these products. A 2019 report by the World Health Organization (WHO) found that 53% of CPCF available in the United Kingdom (UK), 57% in Denmark and 44% in Spain contained total sugars exceeding the WHO’s recommended levels [4]. In the United States (US), most products marketed for IYC were found to contain high sodium levels [5], and the majority of CPCFs available in several Southeast Asian countries contained added sugars or sweeteners [6]. While evidence is not yet conclusive, the consumption of nutritionally inappropriate CPCFs may increase the risk of overweight/obesity later in childhood [7,8]. Moreover, the consumption of unnecessary sweet and salty products at this early age can shape food preferences, establishing habits that persist into later childhood and beyond [9]. Unhealthy products can also displace consumption of more nutritious foods and breastmilk, undermining optimal breastfeeding and complementary feeding practices [10].

CPCF consumption is prevalent in many high-income countries. A cross-sectional study among Australian IYC found that 70% of children aged 12–14 months consumed any CPCF [11], and 63% of children at 2 years of age consumed infant custards and yogurts in a given day [12]. Similarly, the UK Diet and Nutrition Survey of Infants and Young Children reported between 67–72% of infants aged 4–11 months consuming CPCFs for their main meal of the day [13]. In the US, 52% and 33% of infants aged 6–12 months consume commercially produced ‘infant cereals’ and ‘baby finger foods’, respectively [14]. The high consumption of CPCFs in high-income countries can be attributed to various factors. According to a study conducted in the UK, caregivers reported purchasing these products due to concerns over food preparation/food safety, convenience, affordability, and “the way in which commercial snacks provide opportunities for safe development of motor skills, keep infants occupied, and allow them to take part in family rituals” [15]. In the US, caregivers reported primarily purchasing CPCFs for health reasons, [16] while a study of Australian mothers found that some mothers considered CPCFs to be safer than homemade foods due to a lack of confidence in their ability to prepare foods correctly and the age recommendations on the packaging [17]. Although information on CPCF consumption in many upper-middle income countries is limited, use of CPCF products for IYC feeding has been noted. A 2012 national survey in Mexico found that between 12.6–15.0% of infants aged 4–8.9 months consumed commercial baby food fruit, and between 7.3–10.2% of infants aged 6–11.9 months consumed infant cereals at least once a day [18]. The consumption of CPCFs may become increasingly prevalent in upper-middle income countries, given their convenience and expanding availability [1]. In parallel, increasing childhood overweight and obesity poses an urgent global public health concern [19]. Over one-fifth of children below 5 years of age are overweight in Australia, and almost 13% of children 2–5 years of age in the US are obese [20,21]. 

Implementing policies to improve the food environment, including those that clearly communicate the nutritional quality of CPCFs to caregivers, is necessary to curb the growing epidemic of childhood overweight and obesity [22,23]. Front-of-pack nutrition labelling (FOPNL) is one such policy tool that can help rebalance unhealthy food environments [24]. A growing body of evidence indicates that FOPNL can promote healthy diets by facilitating consumers in making healthier food choices [23,25] and driving reformulation by the food industry [26,27]. The FOPNL schemes vary in design, and may be implemented on either a voluntary or mandatory basis. An increasing body of research indicates that interpretive FOPNL, such as “traffic-light” labels adopted in the UK and in the United Arab Emirates (UAE), or the “warning labels” implemented in Chile, Brazil and Mexico, can be effective in stimulating healthier choices at the point of purchase [23,24,25]. Chile provides strong evidence that a mandatory, nutrient-specific warning label results in significant reductions in purchases of unhealthy products [28], and emerging evidence from Mexico also indicates that the national mandatory warning label system has encouraged manufacturers to reduce nutrients of concern, such as sugar, salt and saturated fat [29]. 

Despite the importance of caregivers’ awareness on the nutritional quality of CPCFs, some national FOPNL systems exclude products for IYC or utilize voluntary schemes where CPCF manufacturers are able to opt out of such labelling measures. The exclusion of CPCFs from FOPNL systems, or the adoption of voluntary measures, limit opportunities for communicating nutritional suitability of these products to caregivers and precludes CPCFs from the public health benefits that FOPNL can provide. To better understand what role FOPNL could provide for CPCF labelling, this paper aims to assess the levels of selected nutrients of public health concern in CPCFs available in Australia, Brazil, Chile, Mexico, the UAE, the UK and the USA. The objective of the study was to evaluate CPCFs against national and global FOPNL systems to determine the proportion that would warrant warning/traffic light signs for higher levels of nutrients of public health concern. 

## 2. Materials and Methods

### 2.1. Study Design

A cross-sectional assessment was conducted on CPCF label information from products identified for sale in Australia, Brazil, Chile, Mexico, the UAE, the UK and the US. For this study, CPCFs were defined as products specifically marketed as suitable for feeding older infants and young children, meeting at least one of the following criteria: (1) were recommended for introduction at an age of less than three years; (2) were labelled with the words ‘baby’, ‘infant’, ‘toddler’, ‘young child’ or a synonym; (3) had a label with an image of a child who appeared to be younger than three years of age or who was feeding with a bottle or (4) were in any other way presented as being suitable for children under three years of age [30].

### 2.2. Data Sources and Management

A database of CPCF products available in Australia was purchased from Innova Market Insights (IMI), a market research company. To develop the database, IMI research staff identified newly launched CPCF products through weekly visits to purposively sampled retailers across Australia that were also available for purchase online. After products were identified, label information was extracted by IMI and entered into the database. Photographs of products were also taken and uploaded to the database. Entered product label information was checked by local and regional IMI editors. The database was sourced from IMI in March 2022 and included all newly launched products available in Australia from January 2020 to February 2022. Separately, databases of CPCF products available for purchase in Brazil, Chile, Mexico, the UAE, the UK and the US were purchased from the market research company, Euromonitor International (EI). EI uses their Via platform to extract nutrient content data from online retailers across 40 countries, based on text and label images presented on retailer websites. The six databases were purchased from EI in November 2021 and included all products available from online grocery retailers on 17 November 2021. 

After database purchase from EI and IMI, between January and March 2022, each product’s nutrient content information and ingredient list were cross-checked by study authors against the product photographs (IMI database) or against information presented on retailers’ websites (EI databases) to ensure label information accuracy. The following information was extracted from the databases for use in analysis: brand name, manufacturer, ingredient list, nutrient content per 100 g/serving (energy, total fat, saturated fat, total sugar, added sugar, sodium/salt), reconstitution instructions and serving size. Nutrient contents were calculated per 100 g where contents were declared as per serving. The presence of added sugars/sweeteners were identified within ingredient lists, including sugar, juice (except lemon/lime), sucrose, dextrose, fructose, glucose, maltose, galactose, trehalose, syrup, nectar, honey, malted barley, malt extract and molasses. Nutrient contents per 100 g of reconstituted product were also calculated for the relevant products (i.e., dry/instant cereals and powdered beverages). In these cases, the nutrient contents per 100 g/serving size of the reconstituted product were extracted when available, or these values were calculated following the manufacturer instructions. If the manufacturer instructions for reconstitution were not clear (e.g., quantities of product or type of liquid to be used were imprecise) and the nutrient contents for the reconstituted product were not provided, these products were excluded from analysis. 

### 2.3. FOPNL Analysis

The national FOPNL systems for Brazil, Chile and Mexico were used for the analysis of CPCF products identified in these countries. These are ‘warning sign’ systems that evaluate the content of specific nutrients of public health concern, due to their correlation with diet related NCDs, using a threshold cut-off. For Brazil, CPCF products were flagged for warning signs if foods contained ≥15 g of added sugar, ≥6 g of saturated fat or ≥600 mg of sodium and if liquids contained ≥7.5 g of added sugar, ≥3 g of saturated fat or ≥300 mg of sodium per 100 g/mL product [31]. For Chile, warning signs were flagged for foods containing ≥275 kcal of energy, ≥400 mg of sodium, ≥10 g of total sugar, or ≥4 g of saturated fat and for liquids containing ≥70 kcal of energy, ≥100 mg of sodium, ≥5 g of total sugar or ≥3 g of saturated fat per 100 g/mL of product. While Chile’s legislation exempts CPCFs, unless they contain added sugar [32], all products were run through the FOPNL system for this analysis. For Mexico, warning signs were flagged for foods containing ≥275 kcal of energy per 100 g of product and liquids containing ≥70 kcal of energy per 100 mL of product. Mexican legislation also flags warning signs for liquids containing ≥8 kcal of free sugars per 100 mL of liquids, but this information was not provided on labels in our databases. Additionally, both food and liquid products from Mexico were flagged for warning signs if they contained ≥10% of total energy from added sugars, ≥10% of total energy from saturated fats, ≥1% of total energy from trans fats or ≥1 mg of sodium per kcal (or ≥300 mg sodium per 100 g of product [33]). The national FOPNL systems for the UAE [34] and UK [35] are identical and follow a ‘traffic light’ sign system, which also uses cut-offs, but thresholds differ for low levels (green), moderate levels (amber) and high levels (red) (Table 1). Because the US does not yet have a national FOPNL system, the UAE/UK system was used for these products. While Australia has a FOPNL system, the UAE/UK system was used for these products for two reasons: (1) the Australia FOPNL system (Health Star Rating) is a promotional FOPNL, which has been noted as not appropriate for CPCFs [26], and (2) this system is category-specific (thresholds differ for certain types of foods) and no categories were identified as relevant for CPCF products. 

In 2019, in order to support the implementation of WHA 69.9’s call to identify the inappropriate promotion of CPCFs, the WHO Regional Office for Europe published a nutrient profile model for CPCFs (hereafter referred as the WHO Europe NPM) [4]. The model includes a front-of-pack ‘high sugar’ warning for products that exceed a threshold for the total sugar content. As this is the first FOPNL system developed specifically for CPCFs, products from all seven countries were assessed against the WHO Europe NPM. First, the CPCF product names and ingredients were reviewed, and all products were placed in one of sixteen categories outlined in the WHO Europe NPM [4]. Next, each product was evaluated against a category-specific threshold for the proportion of the energy percentage from the total sugar content. Products that exceeded this threshold were flagged for requiring a high sugar warning on their label.

Analysis was conducted using Stata 14. Descriptive statistics were calculated and summarized, specifically the proportions of products requiring a warning sign or amber/red traffic light sign on their front-of-pack label were calculated.

## 3. Results

### 3.1. Product Characteristics

The final count of CPCF products identified in each country was: 266 in Australia, 41 in Brazil, 73 in Chile, 164 in Mexico, 135 in the United Arab Emirates, 642 in the Unites States and 562 in the United Kingdom (Table 2). Across all seven countries, pureed foods were the most common product category identified, making up 41.5 – 80.8% of CPCFs. Instant cereals were prevalent in Brazil and the UAE (41.5% (*n* = 17) and 39.3% (*n* = 53), respectively), whereas snacks/finger foods were prevalent in Australia (38.7% (*n* = 103)). While CPCF beverage products were identified in four of the seven countries, they were not highly prevalent in these contexts. Nestlé was the manufacturer of the largest number of CPCF products across five of the seven countries, manufacturing 90.2% (*n* = 37) of products in Brazil, 76.7% (*n* = 56) in Chile, 71.3% (*n* = 117) in Mexico, 43.2% (*n* = 243) in the US and 26.7% (*n* = 36) in the UAE (Supplementary Table S1). 

### 3.2. Added Sugars or Sweeteners in CPCF

The addition of added sugars or sweeteners to CPCF products was prevalent in the majority of the countries included in this study (Table 3). Added sugars or sweeteners were noted among almost half (47.4%, *n* = 18) of CPCFs sold in Brazil, over one-third sold in Mexico (38.5%, *n* = 62) and the UAE (34.6%, *n* = 38) and over one-quarter in Australia (27.8%, *n* = 74). Across most countries, the highest proportion of CPCF products containing added sugar/sweetener was noted among the snacks/finger foods category, ranging from 42.8–79.1% of products in six of the seven countries. The exception was Brazil, where none of the three snack/finger food products contained added sugar/sweetener, while all fourteen instant cereal products did. 

### 3.3. WHO Europe Commercial Complementary Food High Sugar FOPNL System

The proportion of CPCF products that required a high sugar warning on their FOP based on the WHO Europe NPM is presented in Table 4. Few instant cereals warranted a high sugar warning, while the majority of pureed foods across all seven countries (ranging from 52.5% in Chile to 100% in Brazil) were identified as having a high sugar content for IYC based on this FOPNL system. Approximately two-thirds of snacks/finger foods in the USA and UAE (66.7%, respectively) and one-third in Australia, the UK and Mexico (39.8%, 39.7%, and 33.3%, respectively) also required a high sugar warning.

### 3.4. Traffic Light FOPNL System—Australia, UAE, UK and USA

The proportion of CPCF products that warranted an amber or red traffic light warning according to national FOPNL systems are presented in Table 5, with the performance of products across each product category detailed in Supplementary Table S2. 

Overall, the proportion of products that warranted a red traffic light sign for high total sugar/salt/saturated fat/total fat content was low: 15.8% (*n* = 42) of CPCFs in Australia, 13.3% (*n* = 18) in the UAE, 7.5% (*n* = 48) in the UK and 6.8% (*n* = 38) in the USA. However, the majority of products warranted an amber traffic light sign for medium total sugar/salt/saturated fat/total fat content: 63.5% (*n* = 169) of CPCFs in Australia, 56.3% (*n* = 76) in the UAE, 59.1% (*n* = 380) in the UK and 78.1% (*n* = 439) in the USA. Across all four countries where the traffic light system was applied, snacks/finger foods was the CPCF product category with the greatest proportion of products warranting an amber or red traffic light; however, the nutrients driving the warning lights for this product category varied. Amber/red warning lights were highly prevalent for the total fat and saturated fat contents among snacks/finger foods in Australia (80.6% and 48.1%, respectively), the UAE (90.9% and 84.6%, respectively) and the UK (80.8% and 42.3%, respectively), but less prevalent among those in the USA (33.3% and 3.9%, respectively). While almost half (49.4%) of snacks/finger foods in the USA warranted an amber/red traffic light for salt, only 13.6–20.2% of snacks/finger foods in Australia, the UAE and the UK contained a medium/high salt content. The total sugar content was medium/high across all product categories in the UAE (50.0% of instant cereals, 77.3% of snacks/finger foods, 83.7% of pureed foods), the UK (81.8% of instant cereals, 44.6% of pureed foods, 67.7% of snacks/finger foods) and the USA (60.0% of instant cereals, 76.7% of pureed foods, and 82.7% of snacks/finger foods). While the total sugar content warranted an amber or red traffic light among 48.5% of pureed foods, 61.1% of snacks/finger foods and 66.6% of beverages in Australia, no instant cereals contained a medium/high total sugar content. 

### 3.5. Warning Sign FOPNL System—Brazil, Chile, and Mexico

**Figure 1:**
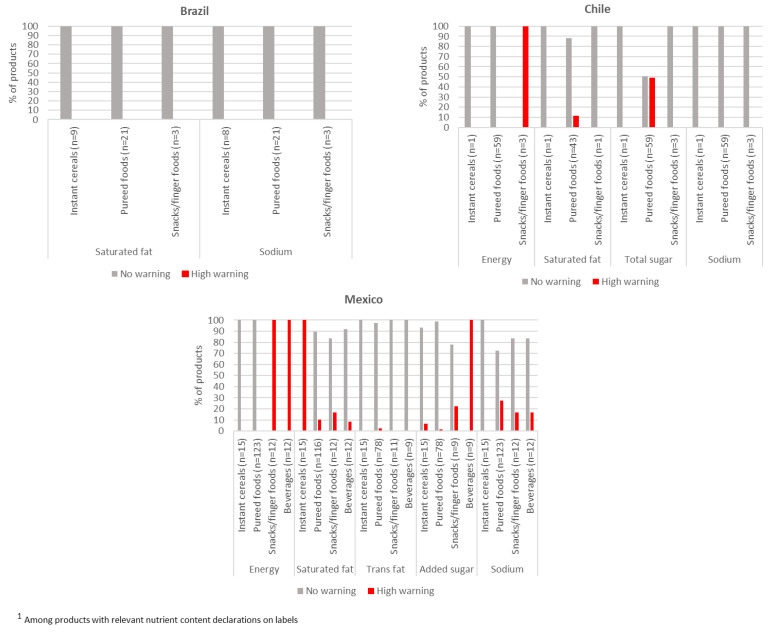
Proportion of commercially produced complementary foods warranting a warning sign ^1^.

The proportion of CPCF products that warranted a warning sign according to national FOPNL systems are presented in Figure 1, with the performance of products across each product category detailed in Supplementary Table S2. Approximately half of CPCF products identified in Chile (45.2%, *n* = 33) and Mexico (46.3%, *n* = 76) warranted a FOP warning sign. In Chile, this was driven by all snacks/finger foods receiving a warning sign for a high energy density and nearly half (49.2%, *n* = 29) of all pureed foods receiving a warning sign for a high total sugar content. In Mexico, all CPCF beverages and snacks/finger foods received a warning sign for a high energy density, and all beverages and 22.2% of snacks/finger foods also received a warning for a high added sugar content. A high saturated fat content was noted among all instant cereals, and a high sodium content was found in 27.6% of pureed foods and 16.7% of snacks/finger foods and beverages. No CPCF products identified in Brazil warranted a FOP warning for saturated fat or sodium. However, none of the CPCFs in Brazil declared an added sugar content and could not be evaluated against this national FOPNL threshold. 

## 4. Discussion

This study assessed the nutrient levels of CPCFs available in Brazil, Chile, Mexico, the USA, the UAE, the UK and Australia against national FOPNL systems and the ‘high sugar’ FOPNL warning based on the WHO Europe NPM. To our knowledge, this is the first study to evaluate nutrient content of CPCFs based on national FOPNL regulations the total sugar content of CPCFs available in Brazil, Chile, Mexico, the USA, the UAE and Australia against the WHO Europe FOPNL high-sugar warning. A substantial proportion of CPCFs, particularly snacks/finger foods and purees, contained excessive levels of total sugar, total fat or saturated fat that would warrant a red/amber traffic light or warning sign on product labels. Additionally, the high prevalence of added sugars and sweeteners identified in CPCFs was concerning. These results indicate that the use of FOPNL among CPCF products could be beneficial to ensure that caregivers are able to make informed decisions when purchasing products for IYC feeding.

Despite the wide consensus on the importance of avoiding added sugars in foods for IYC [30,36,37], many CPCFs available in the seven countries included in this study contained at least one added sugar or sweetener, and the total sugar content among many CPCFs warranted a high sugar warning. Likewise, a study of 2634 CPCF products from ten European countries [38] found that the total energy from sugar ranged from 29% to 44%. Additionally, 45% of CPCFs available in the US were classified as having a ‘high’ sugar content when evaluated in light of American Heart Association recommendations, with more than 20% of their total energy content derived from sugar [39]. For most countries included in this present study, the highest proportion of CPCF products containing added sugars/sweeteners were among the snacks/finger foods category. Previous studies conducted in Europe and the US have also noted widespread use of added sugars/sweeteners among this category of products [5,38]. A study by Devenish et al. found that the most common sources of free sugars consumed by 12–14 months old Australian children were CPCF products, specifically dairy-based desserts and snacks [11]. In the UK, it has been observed that sweet CPCFs dominate the market [40] and contribute significant amounts of total sugar to IYC’s diet [41]. As high sugar intake is associated with an increased risk of overweight and NCDs, the presence of such high levels of sugars in CPCFs is a significant concern [8,42]. Research has shown that an added sugar intake in infancy is associated with a higher added sugar intake in later childhood due to the establishment of taste preferences during this period of early life [43]. Evidence also suggests that high sugary food consumption among children raises their risk of high blood triglycerides and is linked to a higher incidence of dental caries [8]. 

Puréed foods, the most prevalent product category across all seven countries, performed particularly poorly against the total sugar threshold, with 52.2–100% of these products identified as having a high sugar content based on the WHO Europe FOPNL high-sugar warning. Similarly, a study of CPCFs sold in Australia found that fruit purées most commonly contained high sugar levels [44]. A 2016 study of puréed CPCFs in the UK found these products contained a high proportion of fruit and sweet-tasting vegetables, contributing to a high total sugar content, while bitter/less sweet vegetables were rarely used [45]. Despite three-quarters of pureed CPCF products available in Mexico requiring a FOPNL high-sugar warning based on the WHO Europe NPM, only one pureed product received a high-sugar warning sign based on Mexico’s national FOPNL system. The cause of this discrepancy is because the FOPNL system in Mexico considers added sugar content rather than total sugar content [33]. Sugar content of puréed foods is primarily intrinsic and not from added sugars; however, the intense puréeing process used to make these CPCF products releases intrinsic sugars from the cell walls of fruit and vegetables, resulting in readily available free sugars. Given that the high sugar levels of puréed foods is mostly not from added sugars, the assessment of the total sugar content of CPCFs would be preferrable to detect CPCF products presenting excessive sugar levels, as suggested by the WHO Europe NPM [30]. Finally, almost half of the products in Brazil listed at least one added sugar or sweetener in the ingredient list. However, since none of the Brazilian CPCFs declared an added sugar content on the label, they could not be evaluated against the national FOPNL threshold. The lack of an added sugar content declared on the label was likely due to the fact that the Brazilian government announced the final changes for the food labelling regulation at the end of 2020, including the mandatory declaration of the total and added sugar content in grams on the label and the high added sugars FOP warning; however, food manufacturers were not mandated to apply these changes until October 2022 [31]. Additionally, if the food industry produces a large amount of packaging, they may request an extension of the deadline [31].

High/moderate levels of total/saturated fat content were noted among CPCFs in Australia, Mexico, the UK and UAE. A recent study conducted in Europe among 3427 CPCF products [46] found that products belonging to the snacks/finger foods category contained high levels of total and saturated fat, and high levels of total and saturated fats were found in all CPCF products with a less desirable nutrient profile [46]. Fats should be present in adequate amounts in the diet of IYC to provide essential fatty acids and aid absorption of fat-soluble vitamins, as well as to increase energy density, in order to promote cognitive and motor development [1]. However, it is recommended that the total dietary fat intake be gradually reduced throughout childhood, from 40–60% of the total energy intake around 6 months of age to 30–35% of the total energy intake at 24 months, and 25–35% from 2 years onwards [47]. The traffic light FOPNL system applied to Australia, the UK, the US and the UAE sets red and amber traffic lights for a total fat content > 5 g/100 g and >3 g/100 g, respectively, similar to the 3.3–4.5 g/100 g total fat limits present in the Codex Alimentarius standards for cereal-based complementary foods [48]. It is concerning that most of the products that would warrant an amber/red traffic light sign for total/saturated fats belongs to the snacks/finger foods category. As snacks/finger foods are usually consumed by older children [41], consumption of these products would increase rather than decrease the total fat intake as the child gets older. Moreover, given snack/finger foods typical role in the IYC’s diet, lower total fat/saturated fat levels would be appropriate [49].

In this study, all snacks/finger foods available in Chile and Mexico and all CPCF beverages in Mexico received a warning sign for their high energy density. While a low energy density of complementary foods can be problematic, since IYC’s small stomachs mean that they consume relatively small amounts at mealtimes [50], it is essential that energy density comes from nutrient-rich sources [1]. A recent study by Grammatikaki et al. noted that CPCFs presenting a less desirable nutrient profile had considerably higher energy density than those nutritionally suitable for IYC [46]. CPCFs that are energy-dense but nutrient-poor, and tend to be eaten as snacks, may contribute excess energy intake to the diet of IYC [1]. In this present study, the CPCF products identified as having problematically high energy-density were sugar-sweetened beverages and snack products, which global evidence has flagged as products contributing to overweight and obesity among children [51]. If consumed in excess, they could either displace other more nutrient-dense food categories or lead to energy overconsumption and unfavorable gain in body mass [46,52].

While the sodium content of CPCFs was not of particular concern in five of the seven countries, almost half of snacks/finger foods in the US warranted an amber/red traffic light for salt. This finding is consistent with a previous study, which detected high sodium contents in CPCFs available in the US [5].

The prevalence of certain CPCF categories across the seven countries varied widely. Two possible explanations are the differences in cultural norms and dietary preferences towards certain types of CPCFs. For example, in Chile puréed foods may be more popular, while in the UAE dry/instant cereals may be more commonly consumed. Another factor could be differences in the availability or accessibility of certain types of complementary foods in different countries. For example, less mature markets such as Chile, Mexico and Brazil may have fewer product choices and a lower percentage of snack products compared to more mature markets such as UK and Australia. 

A growing number of countries globally have begun the implementation of FOPNL as a strategy to address the high prevalence of overweight and obesity and diet-related NCDs [24,53]. FOPNL aims to support consumers to make healthier food choices at the point of purchase by delivering simplified, at-a-glance nutritional information [22]. The existing guidance, including the Guiding Principles and Framework Manual for FOPNL from the WHO [26], supports government-led action on FOPNL, but does not yet specify that countries should use a particular type of label. In July 2018, the Codex Alimentarius Commission (Codex), a multilateral United Nations body responsible for work on food standards, formally approved ongoing work towards a guideline on FOPNL to respond to the global rise in diet-related NCDs [54]. Codex articulates its standards as the basis for national standards, and its guidance is likely to have a significant impact on the global adoption of FOPNL and influence national policy making. 

Research has shown that FOPNL systems that are mandatory, applied across all packaged food products, and that provide a visual indication of product unhealthfulness, such as a traffic-lights or warning signs, are most effective in discouraging unhealthful purchasing behavior [22,24]. The promotion of CPCFs as products specifically formulated for IYC may lead caregivers to believe they are nutritionally suitable and a healthy option to feed their children. A qualitative study on mothers’ perception of CPCFs conducted in the UK reported that some concerns were raised about the identity of ingredients in these products (e.g., the presence of milk, eggs, gluten, nuts, preservatives, coloring, salt, or hidden ingredients); however, few concerns were raised regarding the nutritional quality (e.g., sugar content) of CPCF products [55]. FOPNL for CPCFs may be an effective means to convey information on the nutritional quality of these products to caregivers. Furthermore, there is some evidence that mandatory FOPNL systems can stimulate companies to reformulate products towards healthier nutrient compositions [24]. In Chile, where a mandatory regulation is in force, increased food reformulation leading to significant reductions in the energy, added sugars and sodium content of pre-packaged foods and beverages after the implementation of the Food Labelling and Advertising Law has been noted [27,56]. 

Consumer studies have been conducted in various contexts to evaluate the acceptability and understanding of FOPNL. In the UK, research exploring consumers’ understanding and use of FOPNL found that a majority of consumers pay attention to FOPNL, and most participants found it useful when trying to choose healthier products [57]. Interestingly, people shopping for their families were most likely to look at FOPNL [57]. Among low and middle-income Mexican consumers, the traffic-light system and warning labels were more accepted and understood compared to Mexican Guideline Daily Allowances, and warning labels were the sole labelling system capable of warning about nutrients of public health concern [58]. In Chile, the law on food labeling and advertising was well known by mothers of diverse socio-economic statuses after five years of implementation, and many mothers expressed that they perceived an important shift toward healthier eating due to the regulation, which may lead to a change in eating social norms [59]. However, to our knowledge, no studies were conducted on the perception and understanding of FOPNL signs on CPCF products and their effects on caregivers’ purchasing decision making. It is noteworthy that there are several differences between the average consumer and caregivers of IYC, and these could lead to different purchasing behaviors. Firstly, caregivers of IYC are responsible for making purchasing decisions on behalf of their child. This could mean that caregivers of IYC prioritize different factors when selecting CPCFs, such as the nutritional content, safety, and convenience, compared to the average consumer, who may prioritize taste, affordability, and other factors. Secondly, caregivers of IYC may have different levels of knowledge and experience related to nutrition and IYC feeding practices compared to the average consumer. They may also have different cultural or social norms that impact their perceptions related to CPCFs. Finally, caregivers of IYC may have a stronger emotional attachment to the child and a desire to provide the best possible nutrition for their growth and development. 

The results of this present study show that a significant proportion of CPCFs in the seven countries under consideration would warrant a FOPNL warning sign for high levels of selected nutrients. Nonetheless, there is currently no explicit guidance on the use of FOPNL among CPCF products. The discussion on whether CPCFs should be explicitly included in FOPNL systems is evolving. On one hand, it has been noted that current national FOPNL systems would not be appropriate for CPCFs since foods for IYC have strict compositional criteria which may differ from the nutrient thresholds set for adults [26]. Addressing the concern of a need for IYC-specific nutrient thresholds for FOPNL, the WHO Euro NPM included a high-sugar FOP warning to provide caregivers guidance when purchasing CPCFs that might contain excessive levels of total sugar [30]. A concern has also be raised that some FOPNL systems may provide promotional advantages to CPCF products [26]. “Promotional” FOPNL systems include overall rating systems (e.g., Nutri-Score and the Health Star Rating) which may provide a positive rating for a product’s nutritional quality and endorsement logos presented on products with favorable nutrient profiles when compared to same-category alternatives. However, during the forty-sixth session of the Codex Alimentarius Committee on FOPNL guidelines development, the Codex agreed not to call for a de facto exclusion of CPCFs from FOPNL systems, leaving it up to nations to decide which products to include in national legislation [60]. 

With the growing availability and use of CPCFs, it is essential to consider the inclusion of CPCF products in national FOPNL. The use of FOPNL among CPCF products has the potential to provide caregivers with a clear and at-a-glance interpretation of the nutritional suitability of products for IYC at the point of purchase and encourage manufacturers’ reformulation of CPCFs to reduce concerning levels of nutrients of public health concern. As underlined by the WHO [26], the adoption of FOPNL systems for CPCFs should avoid positive label endorsements that could result in promotional messages. Warning labels, in contrast, do not carry the risk of creating an inappropriate “health halo” effect on CPCF products [22]. Moreover, FOPNL systems should ensure that nutrient-specific warnings are applied to foods for IYC in line with robust and transparent criteria for nutrient profiling that is tailored to infant and young child dietary requirements, such as the WHO Europe NPM [30]. 

Our study has several limitations and strengths. We obtained a large number of CPCF products from seven countries. The analysis was performed on secondary data on CPCFs sold online provided by Euromonitor and IMI, with all data carefully checked for errors and validated against the pictures of the product label. Moreover, we used the food categories as defined by the WHO Europe NPM to ensure comparability to other studies. However, the study was restricted to the information provided on product labels. If a nutrient was not reported on the label, it could not be evaluated against the FOPNL thresholds. The majority of dry/instant cereals available in the US had to be excluded from the study because they did not report clear reconstitution instructions.

## 5. Conclusions

The food environment plays a critical role in influencing children’s diets. Despite high levels of nutrients of public health concern present in CPCFs, some national FOPNL systems exclude products for IYC in their regulations, or they implement voluntary systems that manufacturers are not mandated to adopt. With the unprecedented availability and accessibility of CPCFs, it is essential to consider whether it is appropriate to include these products in national FOPNL systems. A growing body of evidence suggests that FOPNL provides a mechanism for interpreting and communicating the nutritional quality of food and beverages to consumers, discouraging the selection and purchasing of unhealthy products, and triggering product reformulation. The exclusion of CPCFs from FOPNL systems limits the opportunities for communicating the nutrient content of these products to caregivers and excludes them from the potential public health benefit that FOPNL can provide when products high in nutrients of public health concern are identified. Our results show that including CPCFs in national FOPNL systems can be a useful tool to communicate clearly to caregivers the nutritional quality of these products and ensure informed decision making for child feeding. Future research should investigate how caregivers interpret FOPNL on CPCFs, and how this affects their purchase behavior, as well as the impact of FOPNL systems on manufacturers’ product reformulation.

## Figures and Tables

**Table 1:** FOPNL nutrient threshold cut-offs applied to the UAE, UK, US and Australia.

Per 100 g food		Low	Medium	High	High ^1^
	Fat	≤3 g	>3 g to ≤17.5 g	>17.5 g	>21 g/portion
	Saturated fat	≤1.5 g	>1.5 g to ≤5 g	>5.0 g	>6.0 g/portion
	Total sugar	≤5 g	>5 g to ≤22.5 g	>22.5 g	>27 g/portion
	Salt	≤0.3 g	>0.3 g to ≤1.5 g	>1.5 g	>1.8 g/portion
Per 100 mL drinks		Low	Medium	High	High ^2^
	Fat	≤1.5 g	>1.5 g to ≤8.75 g	>8.75 g	>10.5 g/portion
	Saturated fat	≤0.75 g	>0.75 g to ≤2.5 g	>2.5 g	>3 g/portion
	Total sugar	≤2.5 g	>2.5 g to ≤11.25 g	>11.25 g	>13.5 g/portion
	Salt	≤0.3 g	>0.3 g to ≤0.75	>0.75 g	>0.9 g/portion

^1^ Portion size criteria applied when portion/serving size is greater than 100 g. ^2^ Portion size criteria applied when portion/serving size is greater than 150 mL.

**Table 2:** Summary of commercially produced complementary foods identified.

Country	Dataset Source	Total Products (*n*)	Instant Cereals % (*n*)	Pureed Foods % (*n*)	Snacks/Finger Foods % (*n*)	Beverages % (*n*)
Australia	INNOVA	266	6.8 (18)	52.3 (139)	38.7 (103)	2.2 (6)
Brazil	Euromonitor	41	41.5 (17)	51.2 (21)	7.3 (3)	--
Chile	Euromonitor	73	15.1 (11)	80.8 (59)	4.1 (3)	--
Mexico	Euromonitor	164	10.4 (17)	75.0 (123)	7.3 (12)	7.3 (12)
UAE	Euromonitor	135	39.3 (53)	41.5 (56)	18.5 (25)	0.7 (1)
UK	Euromonitor	643	10.6 (68)	69.0 (444)	20.4 (131)	--
USA	Euromonitor	562	5.0 (28)	79.2 (445)	15.5 (87)	0.3 (2)

**Table 3:** Proportion of commercially produced complementary food products containing added sugar/sweetener, by product categories and country ^1,2^.

Country	All Products	Instant Cereals	Pureed Foods	Snacks/Finger Foods	Beverages
**Australia**	266	18	139	103	6
Contain added sugar/sweetener % (*n*)	27.8 (74)	0.0 (0)	15.8 (22)	49.5 (51)	16.7 (1)
**Brazil**	38	14	21	3	--
Contain added sugar/sweetener % (*n*)	47.4 (18)	100.0 (14)	19.1 (4)	0.0 (0)	
**Chile**	69	11	55	3	--
Contain added sugar/sweetener % (*n*)	7.3 (5)	27.3 (3)	0.0 (0)	66.7 (2)	
**Mexico**	161	15	123	11	12
Contain added sugar/sweetener % (*n*)	38.5 (62)	33.3 (5)	31.7 (39)	54.6 (6)	100.0 (12)
**United Arab Emirates**	110	47	43	19	1
Contain added sugar/sweetener % (*n*)	34.6 (38)	36.2 (17)	11.6 (5)	79.0 (15)	100.0 (1)
**United Kingdom**	642	67	444	131	--
Contain added sugar/sweetener % (*n*)	15.0 (96)	11.9 (8)	7.2 (32)	42.8 (56)	
**United States of America**	556	28	440	86	2
Contain added sugar/sweetener % (*n*)	20.2 (112)	17.9 (5)	8.9 (39)	79.1 (68)	0.0 (0)

^1^ Among products that provided ingredient lists on labels, ^2^ the following were considered as having added sugar/sweetener: sugar, juice (except lemon/lime), sucrose, dextrose, fructose, glucose, maltose, galactose, trehalose, syrup, nectar, honey, malted barley, malt extract and molasses.

**Table 4:** Proportion of commercially produced complementary foods warranting a WHO Europe high sugar front-of-pack warning, by product category ^1^.

	Dry/Instant Cereals ^2^ %	Pureed Foods ^3^ %	Snack/Finger Foods ^4^ %
Australia	0.0%	67.4%	39.8%
Brazil	0.0%	100.0%	0.0%
Chile	0.0%	52.5%	0.0%
Mexico	5.9%	74.8%	33.3%
United Arab Emirates (UAE)	2.5%	92.3%	60.0%
United States (US)	0.0%	86.0%	66.7%
United Kingdom (UK)	0.0%	62.6%	39.7%

^1^ Only products declaring sugar content on the labels could be included in the analysis. ^2^ Australia: *n* = 18; Brazil: *n* = 1; Chile: *n* = 11; Mexico: *n* = 17; UAE: *n* = 40; USA: *n* = 28; UK: *n* = 67. ^3^ Australia: *n* = 138; Brazil: *n* = 5; Chile: *n* = 59; Mexico: *n* = 123; UAE: *n* = 52; USA: *n* = 442; UK: *n* = 444. ^4^ Australia: *n* = 103; Brazil: *n* = 1; Chile: *n* = 3; Mexico: *n* = 12; UAE: *n* = 25; USA: *n* = 81; UK: *n* = 131.

**Table 5:** Proportion of commercially produced complementary foods warranting a green, amber or red traffic light.

		Total Fat				Saturated Fat				Total Sugar				Salt			
		Green (%)	Amber (%)	Red (%)	Missing (*n*) ^1^	Green (%)	Amber (%)	Red (%)	Missing (*n*) ^1^	Green (%)	Amber (%)	Red (%)	Missing (*n*) ^1^	Green (%)	Amber (%)	Red (%)	Missing (*n*) ^1^
Australia	Dry/instant cereals (*n* = 18)	100.0	0.0	0.0	2	100.0	0.0	0.0	10	100.0	0.0	0.0	2	100.0	0.0	0.0	2
	Pureed foods (*n* = 139)	72.6	16.7	0.7	1	82.2	16.8	1.0	38	51.5	47.8	0.7	1	100.0	0.0	0.0	1
	Snacks/finger foods (*n* = 103)	19.4	69.9	10.7	0	52.0	31.4	16.7	1	38.8	32.0	29.1	0	79.8	20.2	0.0	0
	beverages (*n* = 6)	66.7	33.3	0.0	0	50.0	0.0	50.0	0	33.3	33.3	33.3	0	100.0	0.0	0.0	0
United Arab Emirates	Dry/instant cereals (*n* = 53)	80.0	20.0	0.0	23	95.5	4.5	0.0	31	50.0	50.0	0.0	35	100.0	0.0	0.0	23
	Pureed foods (*n* = 56)	94.0	6.0	0.0	6	85.5	15.0	0.0	36	14.3	83.7	2.0	7	95.7	0.0	4.3	9
	Snacks/finger foods (*n* = 25)	9.1	81.8	9.1	3	15.4	61.5	23.1	12	22.7	31.8	45.5	3	86.4	13.6	0.0	3
	Beverages (*n* = 1)	100.0	0.0	0.0	0	-	-	-	1	-	-	-	1	100.0	0.0	0.0	0
United Kingdom	Dry/instant cereals (*n* = 68)	61.4	38.6	0.0	24	95.4	4.6	0.0	24	18.2	81.8	0.0	24	100.0	0.0	0.0	24
	Pureed foods (*n* = 444)	89.4	10.6	0.0	0	88.7	11.3	0.0	0	55.2	44.6	0.2	0	99.8	0.2	0.0	15
	Snacks/finger foods (*n* = 131)	19.2	73.9	6.9	1	57.7	33.1	9.2	1	32.3	40.0	27.7	1	80.0	19.2	0.8	1
United States	Dry/instant cereals (*n* = 28)	100.0	0.0	0.0	23	100.0	0.0	0.0	26	40.0	60.0	0.0	23	100.0	0.0	0.0	23
	Pureed foods (*n* = 445)	95.1	4.7	0.2	0	97.2	2.8	0.0	127	22.8	76.7	0.5	7	97.1	2.9	0.0	0
	Snacks/finger foods (*n* = 87)	66.7	20.7	12.6	0	95.1	1.6	3.3	26	17.3	49.4	33.3	6	50.6	48.3	1.1	0
	Beverages (*n* = 2)	100.0	0.0	0.0	0	100.0	0.0	0.0	0	100.0	0.0	0.0	0	100.0	0.0	0.0	0

^1^ missing nutrient content declaration on labels.

## Data Availability

Restrictions apply to the availability of these data. Data were obtained from Euromonitor International and Innova Market Insights and can be accessed from these third parties at https://www.euromonitor.com/ and https://www.innovamarketinsights.com/ (accessed on 30 January 2023).

## Supplementary Materials

The following supporting information can be downloaded at: https://www.mdpi.com/article/10.3390/nu15071629/s1, Supplementary Table S1: Commercially produced complementary food characteristics, by country1; Supplementary Table S2: (a): Front-of-pack labelling results for commercially produced complementary foods available online in Australia (*n* = 266); (b): Front-of-pack labelling results for commercially produced complementary foods available online in the United Arab Emirates (*n* = 135); (c): Front-of-pack labelling results for commercially produced complementary foods available online in the United Kingdom (*n* = 643); (d): Front-of-pack labelling results for commercially produced complementary foods available online in the United States (*n* = 562); (e): Front-of-pack labelling results for commercially produced complementary foods available online in Brazil (*n* = 41); (f): Front-of-pack labelling results for commercially produced complementary foods available online in Chile (*n* = 73); (g): Front-of-pack labelling results for commercially produced complementary foods available online in Mexico (*n* = 170).
