# Supplementary material for: Prevalence of Front-of-Pack Warning Signs among Commercial Complementary Foods in Seven High and Upper Middle-Income Countries
Source: Nutrients. 2023 Mar 27;15(7):1629. doi: 10.3390/nu15071629 (PMC10096843; doi:10.3390/nu15071629)
Supplement: Supplementary file 1 [file nutrients-15-01629-s001.zip › nutrients-2222952-supplementary/Supplementary Table S1.pdf]

Supplementary Table S1. Commercially produced complementary food characteristics, by country[1].

	AUSTRALIA (N=266)	
**Manufacturer**	**Product category**	**% (n)**
Pz Cussons	Pureed foods (n=16)<br>Snacks/finger foods (n=12)	10.5 (28)
Heinz	Pureed foods (n=26)<br>Snacks/finger foods (n=1)	10.2 (27)
Every Bite Counts	Pureed foods (n=11)<br>Snacks/finger foods (n=14)	9.4 (25)
Coles	Pureed foods (n=17)<br>Snacks/finger foods (n=4)	7.9 (21)
Nourish Foods	Pureed foods (n=1)<br>Snacks/finger foods (n=17)<br>Beverages (n=1)	7.1 (19)
The Kids Food Company	Snacks/finger foods	6.0 (16)
Bubs Australia	Instant cereals (n=2)<br>Pureed foods (n=9)<br>Snacks/finger foods (n=3)	5.3 (14)
Only Organic	Pureed foods (n=4)<br>Snacks/finger foods (n=8)	4.5 (12)
Tiny Turtle Baby Food	Pureed foods	4.1 (11)
The Nourishing Company	Pureed foods	3.8 (10)
Annabel Karmel	Pureed foods (n=2)<br>Snacks/finger foods (n=7)	3.4 (9)
Bellamy's Organic	Instant cereals (n=2)<br>Pureed foods (n=5)	2.6 (7)
Funch	Instant cereals (n=3)<br>Pureed foods (n=4)	2.6 (7)
Max Biocare	Pureed foods	2.6 (7)
Lactalis	Pureed foods	2.3 (6)
Woolworths	Pureed foods	2.3 (6)
Little Quacker	Snacks/finger foods	1.9 (5)
Nestlé	Instant cereals	1.9 (5)
Want Want	Snacks/finger foods	1.9 (5)
Mavella Superfoods	Pureed foods (n=1)<br>Beverages (n=3)	1.5 (4)
Sprout Foods	Snacks/finger foods	1.5 (4)
Sweetpea Foods	Instant cereals	1.5 (4)
Aldi	Snacks/finger foods	1.1 (3)
Health Lab	Snacks/finger foods	1.1 (3)
Parmalat	Pureed foods	1.1 (3)
Nutra Organics	Snacks/finger foods (n=1)<br>Beverages (n=1)	0.8 (2)
Asahi	Beverages	0.4 (1)
Ricegrowers	Instant cereals	0.4 (1)
Sun Rice	Instant cereals	0.4 (1)

BRAZIL (N=41)		
**Manufacturer**	**Product category**	**% (n)**
Nestlé	Instant cereals (n=14)<br>Pureed foods (n=20)<br>Snacks/finger foods (n=3)	90.2 (37)
Unilever Group	Instant cereals	7.3 (3)
Pic-Me	Pureed foods	2.4 (1)

CHILE (N=73)		
**Manufacturer**	**Product category**	**% (n)**
Nestlé	Instant cereals (n=10)<br>Pureed foods (46)	76.7 (56)
Ama Time	Pureed foods	15.1 (11)
Fruselva América	Instant cereals (n=1)<br>Pureed foods (n=1)<br>Snacks/finger foods (n=1)	4.1 (3)
Hot-Kid	Snacks/finger foods	1.4 (1)
Empresas Carozzi	Pureed foods	1.4 (1)
Want Want	Snacks/finger foods	1.4 (1)

MEXICO (N=164)		
**Manufacturer**	**Product category**	**% (n)**
Nestlé	Instant cereals (n=16)<br>Pureed foods (n=82)<br>Snacks/finger foods (n=10)<br>Beverages (n=9)	71.3 (117)
Heinz	Pureed foods (n=27)<br>Snacks/finger foods (n=1)<br>Beverages (n=3)	18.9 (31)
Danone	Pureed foods	8.5 (14)
Hain Celestial Group	Instant cereals	0.6 (1)
Walmart Inc	Snacks/finger foods	0.6 (1)

UNITED ARAB EMIRATES (N=135)		
**Manufacturer**	**Product category**	**% (n)**
Nestlé	Instant cereals (n=23)<br>Pureed foods (n=9)<br>Snacks/finger foods (n=4)	26.7 (36)
Hipp	Instant cereals (n=3)<br>Pureed foods (n=10)<br>Snacks/finger foods (n=2)	11.1 (15)
DMK Group	Instant cereals (n=10)<br>Pureed foods (n=1)	8.2 (11)
Hero Group	Instant cereals (n=3)<br>Pureed foods (n=3)<br>Snacks/finger foods (n=3)	7.4 (10)

	Beverages (n=1)<br>Pureed foods (n=9)<br>Snacks/finger foods (n=1)	7.4 (10)
Hain Celestial Group		
Heinz	Snacks/finger foods	6.7 (9)
Holle Baby Food	Instant cereals (n=8)<br>Snacks/finger foods (n=1)	6.7 (9)
Lat Eko Food	Pureed foods	6.7 (9)
The Kids Food Company	Pureed foods (n=1)<br>Snacks/finger foods (n=5)	4.4 (6)
Nutty Nuts	Instant cereals (n=4)<br>Other (n=1)	3.7 (5)
Piccolo Foods Ltd	Pureed foods	3.7 (5)
Carrefour	Pureed foods	3.0 (4)
Danone	Instant cereals	0.7 (1)
Danube Foods Group	Pureed foods	0.7 (1)
Fruchtbar	Pureed foods	0.7 (1)
Organic Baby Food Company	Instant cereals	0.7 (1)
Produsa	Pureed foods	0.7 (1)
Windmill Organics Ltd	Pureed foods	0.7 (1)

UNITED KINGDOM (N=643)

Manufacturer	Product category	% (n)
Hain Celestial Group	Instant cereals (n=3)<br>Pureed foods (n=123)<br>Snacks/finger foods (n=28)	24.0 (154)
Heinz	Instant cereals (n=14)<br>Pureed foods (n=57)<br>Snacks/finger foods (n=11)	12.8 (82)
Danone	Instant cereals (n=17)<br>Pureed foods (n=59)<br>Snacks/finger foods (n=1)	12.0 (77)
Hipp	Instant cereals (n=7)<br>Pureed foods (n=54)	9.5 (61)
Hero Group	Instant cereals (n=6)<br>Pureed foods (n=9)<br>Snacks/finger foods (n=30)	7.0 (45)
The Kids Food Company	Pureed foods (n=4)<br>Snacks/finger foods (n=35)	6.1 (39)
ASDA Little Angels	Pureed foods (n=23)<br>Snacks/finger foods (n=7)	4.7 (30)
Piccolo Foods Ltd	Instant cereals (n=1)<br>Pureed foods (n=26)<br>Snacks/finger foods (n=2)	4.5 (29)
Little Freddie Products	Pureed foods (n=23)<br>Snacks/finger foods (n=5)	4.4 (28)
Sainsbury	Instant cereals (n=5)<br>Pureed foods (n=21)	4.2 (27)

	Snacks/finger foods (n=1)	
Babease Ltd	Pureed foods (n=17)<br>Snacks/finger foods (n=2)	3.0 (19)
Tesco	Pureed foods (n=12)<br>Snacks/finger foods (n=1)	2.0 (13)
Annabel Karmel	Pureed foods	1.4 (9)
Aptamil	Instant cereals	1.2 (8)
Nestlé	Instant cereals (n=4)<br>Snacks/finger foods (n=3)	1.1 (7)
H&H Group	Pureed foods (n=5)<br>Snacks/finger foods (n=1)	0.9 (6)
Lotus Bakeries	Snacks/finger foods	0.6 (4)
Walgreens Boots Alliance Inc	Instant cereals	0.5 (3)
Clearspring Ltd	Pureed foods	0.2 (1)
Holle Baby Food	Pureed foods	0.2 (1)

UNITED STATES (N=562)

Manufacturer	Product category	% (n)
Nestlé	Instant cereals (n=23)<br>Pureed foods (n=175)<br>Snacks/finger foods (n=43)<br>Beverages (n=2)	43.2 (243)
Hero Group	Pureed foods (n=88)<br>Snacks/finger foods (n=2)	16.0 (90)
Danone	Instant cereals (n=2)<br>Pureed foods (n=60)<br>Snacks/finger foods (n=20)	14.6 (82)
Campbell Soup Co	Pureed foods (n=42)<br>Snacks/finger foods (n=4)	8.2 (46)
Hain Celestial Group	Instant cereals (n=3)<br>Pureed foods (n=39)<br>Snacks/finger foods (n=4)	8.2 (46)
Simple Truth Organic	Pureed foods (n=13)<br>Snacks/finger foods (n=2)	2.7 (15)
Happy Tot	Pureed foods (n=11)<br>Snacks/finger foods (n=1)	2.1 (12)
Peter Rabbit Organics	Pureed foods	1.3 (7)
Once Upon a Farm	Pureed foods	0.9 (5)
Hampden Trading Pty Ltd	Snacks/finger foods	0.7 (4)
Kerry Group	Pureed foods	0.5 (3)
Walmart Inc	Snacks/finger foods	0.5 (3)
Want Want	Snacks/finger foods	0.5 (3)
Earth's Best	Snacks/finger foods	0.2 (1)
Love My Veggies	Pureed foods	0.2 (1)
Tippy Toes	Pureed foods	0.2 (1)

[1]Values are presented as percentage of products per location (number of products per location).
