# Supplementary material for: Prevalence of Front-of-Pack Warning Signs among Commercial Complementary Foods in Seven High and Upper Middle-Income Countries
Source: Nutrients. 2023 Mar 27;15(7):1629. doi: 10.3390/nu15071629 (PMC10096843; doi:10.3390/nu15071629)
Supplement: Supplementary file 1 [file nutrients-15-01629-s001.zip › nutrients-2222952-supplementary/Supplementary Table S2.pdf]

Supplementary Table S2a. Front-of-pack labelling results for commercially produced complementary foods available online in Australia (N=266).

FOP label outcome	Instant cereals	Pureed foods	Finger foods/snacks	Beverages
Total fat	n=16	n=138	n=103	n=6
Low (green)	100.0 (16)	82.6 (114)	19.4 (20)	66.7 (4)
Medium (amber)	0.0 (0)	16.7 (23)	69.9 (72)	33.3 (3)
High (red)	0.0 (0)	0.7 (1)	10.7 (11)	0.0 (0)
Saturated fat	n=8	n=101	n=102	n=6
Low (green)	100.0 (8)	82.2 (83)	52.0 (53)	50.0 (3)
Medium (amber)	0.0 (0)	16.8 (17)	31.4 (32)	0.0 (0)
High (red)	0.0 (0)	1.0 (1)	16.7 (17)	50.0 (3)
Total sugar	n=16	n=138	n=103	n=6
Low (green)	100.0 (16)	51.5 (71)	38.8 (40)	33.3 (2)
Medium (amber)	0.0 (0)	47.8 (66)	32.0 (33)	33.3 (2)
High (red)	0.0 (0)	0.7 (1)	29.1 (30)	33.3 (2)
Salt	n=16	n=134	n=99	n=6
Low (green)	100.0 (16)	100.0 (134)	79.8 (79)	100.0 (6)
Medium (amber)	0.0 (0)	0.0 (0)	20.2 (20)	0.0 (0)
High (red)	0.0 (0)	0.0 (0)	0.0 (0)	0.0 (0)

Supplementary Table S2b. Front-of-pack labelling results for commercially produced complementary foods available online in the United Arab Emirates (N=135).

FOP label outcome	Instant cereals	Pureed foods	Finger foods/snacks	Beverages
Total fat	n=30	n=50	n=22	n=1
Low (green)	80.0 (24)	94.0 (47)	9.1 (2)	100.0 (1)
Medium (amber)	20.0 (6)	6.0 (3)	81.8 (18)	0.0 (0)
High (red)	0.0 (0)	0.0 (0)	9.1 (2)	0.0 (0)
Saturated fat	n=22	n=20	n=13	n=0
Low (green)	95.5 (21)	85.0 (17)	15.4 (2)	--
Medium (amber)	4.5 (1)	15.0 (3)	61.5 (8)	--
High (red)	0.0 (0)	0.0 (0)	23.1 (3)	--
Total sugar	n=18	n=49	n=22	n=0
Low (green)	50.0 (9)	14.3 (7)	22.7 (5)	--
Medium (amber)	50.0 (9)	83.7 (41)	31.8 (7)	--
High (red)	0.0 (0)	2.0 (1)	45.5 (10)	--
Salt	n=30	n=47	n=22	n=1
Low (green)	100.0 (0)	95.7 (45)	86.4 (19)	100.0 (0)
Medium (amber)	0.0 (0)	0.0 (0)	13.6 (3)	0.0 (0)
High (red)	0.0 (0)	4.3 (2)	0.0 (0)	0.0 (0)

Supplementary Table S2c. Front-of-pack labelling results for commercially produced complementary foods available online in the United Kingdom (N=643).

FOP label outcome	Instant cereals	Pureed foods	Finger foods/snacks
Total fat	n=44	n=444	n=130
Low (green)	61.4 (27)	89.4 (397)	19.2 (25)
Medium (amber)	38.6 (17)	10.6 (47)	73.9 (96)
High (red)	0.0 (0)	0.0 (0)	6.9 (9)
Saturated fat	n=44	n=444	n=130
Low (green)	95.4 (42)	88.7 (394)	57.7 (75)
Medium (amber)	4.6 (2)	11.3 (50)	33.1 (43)
High (red)	0.0 (0)	0.0 (0)	9.2 (12)
Total sugar	n=44	n=444	n=130
Low (green)	18.2 (8)	55.2 (245)	32.3 (42)
Medium (amber)	81.8 (36)	44.6 (198)	40.0 (52)
High (red)	0.0 (0)	0.2 (1)	27.7 (36)
Salt	n=44	n=429	n=130
Low (green)	100.0 (44)	99.8 (428)	80.0 (104)
Medium (amber)	0.0 (0)	0.2 (1)	19.2 (25)
High (red)	0.0 (0)	0.0 (0)	0.8 (1)

Supplementary Table S2d. Front-of-pack labelling results for commercially produced complementary foods available online in the United States (N=562).

FOP label outcome	Instant cereals	Pureed foods	Finger foods/snacks	Beverages
Total fat	n=5	n=444	n=87	n=2
Low (green)	100.0 (5)	95.1 (442)	66.7 (58)	100.0 (2)
Medium (amber)	0.0 (0)	4.7 (21)	20.7 (18)	0.0 (0)
High (red)	0.0 (0)	0.2 (1)	12.6 (11)	0.0 (0)
Saturated fat	n=2	n=318	n=61	n=2
Low (green)	100.0 (2)	97.2 (309)	95.1 (58)	100.0 (2)
Medium (amber)	0.0 (0)	2.8 (9)	1.6 (1)	0.0 (0)
High (red)	0.0 (0)	0.0 (0)	3.3 (2)	0.0 (0)
Total sugar	n=5	n=438	n=81	n=2
Low (green)	40.0 (2)	22.8 (100)	17.3 (14)	100.0 (2)
Medium (amber)	60.0 (3)	76.7 (336)	49.4 (40)	0.0 (0)
High (red)	0.0 (0)	0.5 (2)	33.3 (27)	0.0 (0)
Salt	n=5	n=445	n=87	n=2
Low (green)	100.0 (5)	97.1 (432)	50.6 (44)	100.0 (2)
Medium (amber)	0.0 (0)	2.9 (13)	48.3 (42)	0.0 (0)
High (red)	0.0 (0)	0.0 (0)	1.1 (1)	0.0 (0)

Supplementary Table S2e. Front-of-pack labelling results for commercially produced complementary foods available online in Brazil (N=41).

FOP label outcome	Instant cereals	Pureed foods	Finger foods/snacks
Added sugar	NA	NA	NA
No warning sign	NA	NA	NA
High warning sign	NA	NA	NA
Saturated fat	n=9	n=21	n=3
No warning sign	100.0 (9)	100.0 (21)	100.0 (3)
High warning sign	0.0 (0)	0.0 (0)	0.0 (0)
Sodium	n=8	n=21	n=3
No warning sign	100.0 (8)	100.0 (21)	100.0 (3)
High warning sign	0.0 (0)	0.0 (0)	0.0 (0)

NA: data not available

Supplementary Table S2f. Front-of-pack labelling results for commercially produced complementary foods available online in Chile (N=73).

FOP label outcome	Instant cereals	Pureed foods	Finger foods/snacks
Energy	n=1	n=59	n=3
No warning sign	100.0 (1)	100.0 (59)	0.0 (0)
High warning sign	0.0 (0)	0.0 (0)	100.0 (3)
Saturated fat	n=1	n=43	n=1
No warning sign	100.0 (0)	88.4 (38)	100.0 (0)
High warning sign	0.0 (0)	11.6 (5)	0.0 (0)
Total sugar	n=1	n=59	n=3
No warning sign	100.0 (1)	50.8 (30)	100.0 (3)
High warning sign	0.0 (0)	49.2 (29)	0.0 (0)
Sodium	n=1	n=59	n=3
No warning sign	100.0 (1)	100.0 (59)	100.0 (3)
High warning sign	0.0 (0)	0.0 (0)	0.0 (0)

Supplementary Table S2g. Front-of-pack labelling results for commercially produced complementary foods available online in Mexico (N=170).

FOP label outcome	Instant cereals	Pureed foods	Finger foods/snacks	Beverages
Energy	n=15	n=123	n=12	n=12
No warning sign	100.0 (15)	100.0 (123)	0.0 (0)	0.0 (0)
High warning sign	0.0 (0)	0.0 (0)	100.0 (12)	100.0 (12)
Saturated fat	n=15	n=116	n=12	n=12
No warning sign	0.0 (0)	89.7 (104)	83.3 (10)	91.7 (11)
High warning sign	100.0 (15)	10.3 (12)	16.7 (2)	8.3 (1)
Trans fat	n=15	n=78	n=11	n=9
No warning sign	100.0 (15)	97.4 (76)	100.0 (11)	100.0 (9)
High warning sign	0.0 (0)	2.6 (2)	0.0 (0)	0.0 (0)
Added sugar	n=15	n=78	n=9	n=9
No warning sign	93.3 (14)	98.7 (77)	77.8 (7)	0.0 (0)
High warning sign	6.7 (1)	1.3 (1)	22.2 (2)	100.0 (9)
Sodium	n=15	n=123	n=12	n=12
No warning sign	100.0 (15)	72.4 (89)	83.3 (10)	83.3 (10)
High warning sign	0.0 (0)	27.6 (34)	16.7 (2)	16.7 (2)
